# Trends and associated factors of animal source foods consumption among children aged 6–23 months in Bangladesh: evidence from four consecutive national surveys

**DOI:** 10.1017/jns.2025.7

**Published:** 2025-02-21

**Authors:** Rafid Hassan, Md Jarif Mahbub, Masum Ali, Teresia Mbogori, Md Ruhul Amin

**Affiliations:** 1 Institute of Nutrition and Food Science, University of Dhaka, Dhaka, Bangladesh; 2 Nutrition Research Division, International Centre for Diarrhoeal Disease Research, Bangladesh (icddr,b), Dhaka, Bangladesh; 3 Department of Nutrition and Food Engineering, Faculty of Health and Life Sciences, Daffodil International University, Dhaka, Bangladesh; 4 Poverty, Gender, and Inclusion, International Food Policy Research Institute (IFPRI), Dhaka, Bangladesh; 5 Department of Nutrition and Health Science, Ball State University, Muncie, IN, USA

**Keywords:** Animal source foods, Bangladesh, BDHS, Children, Complementary feeding, AIC, Akaike’s information criterion, ANC, antenatal care, AOR, adjusted odds ratios, ASF, animal source foods, BDHS, Bangladesh demographic and health survey, BIC, Bayesian information criterion, COR, crude odds ratios, DHS, demographic and health survey, ICC, intra-class correlation coefficient, LMICs, low- or middle-income countries, MDD, minimum dietary diversity, MOR, median odds ratio, PCV, proportional change in variance, VIF, variance inflation factor

## Abstract

Animal source foods (ASF) are nutrient-dense and essential for the growth and development of children. The Bangladesh Demographic and Health Survey (BDHS) 2022 reported that approximately two-thirds of children aged 6–23 months consumed eggs/flesh foods. However, overall consumption patterns, trends, and factors influencing ASF intake among children in Bangladesh were not well-documented. Therefore, the study aimed to assess the trends and associated factors of ASF consumption among children aged 6–23 months in Bangladesh. A total of 9401 children were extracted from four consecutive BDHS (2011, 2014, 2017/18, and 2022). The Cochran-Armitage test was conducted to assess the trends in ASF consumption, while a two-stage multilevel mixed-effects logistic regression was performed to identify the associated factors. The consumption of ASF significantly increased to 79.1% in 2017/18 from 67% in 2011 but decreased to 73.3% in 2022. ASF consumption was found to be higher among children whose mothers were educated (AOR = 1.60, 95% CI = 1.30–1.98), employed in either agricultural (AOR = 1.27, 95% CI = 1.04–1.54) or non-agricultural (AOR = 1.36, 95% CI = 1.07–1.72) activities, pregnant (AOR = 2.54, 95% CI = 1.66–3.87), had received ANC 1–3 times (AOR = 1.43, 95% CI = 1.20–1.72) or ≥4 times (AOR = 1.59, 95% CI = 1.29–1.95), and was exposed to media (AOR = 1.21, 95% CI = 1.04–1.39). Furthermore, consumption increased with increasing the age of children, and the wealth of their families. However, children who experienced illness were less likely to consume ASF (AOR = 0.76, 95% CI = 0.68–0.86). The recent declines in ASF consumption emphasize the need for targeted interventions to increase ASF consumption among children in Bangladesh.

## Introduction

Animal source foods (ASF) refer to food products that come from animals, including meat, fish, poultry, dairy products, and eggs.^(1)^ These foods are rich in protein of high biological value and essential micronutrients such as iron, zinc, vitamin B_12_, calcium, vitamin A, vitamin D, and omega-3 fatty acids.^(2–4)^ The consumption of ASF, thus, can help prevent micronutrient deficiencies, which are common in young children of low- or middle-income countries (LMICs) like Bangladesh^(5,6)^ and can lead to severe health problems if left untreated.^(7–11)^ The nutrients present in ASF are often more easily absorbed and utilized by the body than plant-based sources.^(2,12)^ Introducing a variety of ASF in the diet of children can promote dietary diversity, which is essential for obtaining a broad spectrum of nutrients and reducing the risk of nutritional deficiencies.^(13,14)^

Despite numerous efforts to combat child undernutrition, it remains a significant global public health issue. In 2020, approximately 149 million (22.0%) of under 5 children were suffering from stunting, while 45 million (6.7%) were wasted.^(15)^ Early childhood undernutrition has severe impacts on physical and cognitive development, leading to poorer academic performance, increased health problems, and contributing to nearly half (45%) of all under-five deaths.^(7–11)^ During the prenatal phase and the initial 24 months after birth, there exists a critical 1000-day timeframe when proper nutrition, including recommended breastfeeding and complementary feeding practices, is essential for the well-being of infants and young children.^(7)^ ASF plays a crucial role in providing essential nutrients needed for growth and development during this period. These nutrients are also vital for brain development, immune function, vision, and overall health.^(1,2,4,10,16)^ ASF, introduced as complementary foods after six months of age, are energy-dense, making them particularly beneficial for young children with small appetites or those at risk of malnutrition.^(3,16)^

Globally, a wide regional variation was observed in ASF consumption with the highest consumption recorded in Central or Eastern Europe and Central Asia, while the lowest was in South Asia (where Bangladesh is located). However, Bangladeshis were the fourth lowest consumer of ASF, usually less than 1 serving daily.^(17)^ According to a recent demographic and health survey, about two-thirds of children aged 6–23 months in Bangladesh consumed eggs or flesh food.^(18)^ Another study in rural Bangladesh reported that the consumption of flesh foods, eggs, and dairy among them was 38.0%, 19.7%, and 37.0% respectively.^(19)^ Besides, the diet of Bangladeshi children lacks diversity indicating a cereals-based monotonous diet with limited nutrient-rich ASF.^(18,20)^

Over the past decade, there has been a tremendous improvement in child nutritional status in Bangladesh. The prevalence of stunting among children aged 6–23 months declined from 35% in 2007 to 22% in 2022.^(18,21)^ Likewise, the percentage of underweight children decreased from 35% in 2007 to 18% in 2022.^(18,21)^ Additionally, wasting, which had remained critically high at 21% in 2007, and decreased to 9% in 2022.^(18,21)^ Despite this success, micronutrient deficiency remains a public health concern among children and women in Bangladesh.^(5)^ Approximately 45% of children aged 6–59 months were deficient in zinc, 40% in vitamin D, 24% in calcium, 21% in vitamin A, and 11% in iron.^(22)^ These micronutrients are commonly available through consuming ASF.^(4)^

Due to the high energy and micronutrient density in ASF, their consumption is important in addressing malnutrition among children. However, there was a scarcity of evidence on overall ASF consumption, trends, and the factors influencing ASF consumption among children in Bangladesh. Studies conducted in other countries identified various factors affecting ASF consumption, such as household livelihood profiles, religion, culture, knowledge, women empowerment, maternal nutritional status, region, social safety net programme, seasons, and the age of the children.^(20,23–25)^ Therefore, this study aimed to investigate the trends and the associated factors of ASF consumption among children aged 6–23 months in Bangladesh.

## Methods

### Data sources and sampling

**Fig. 1.**
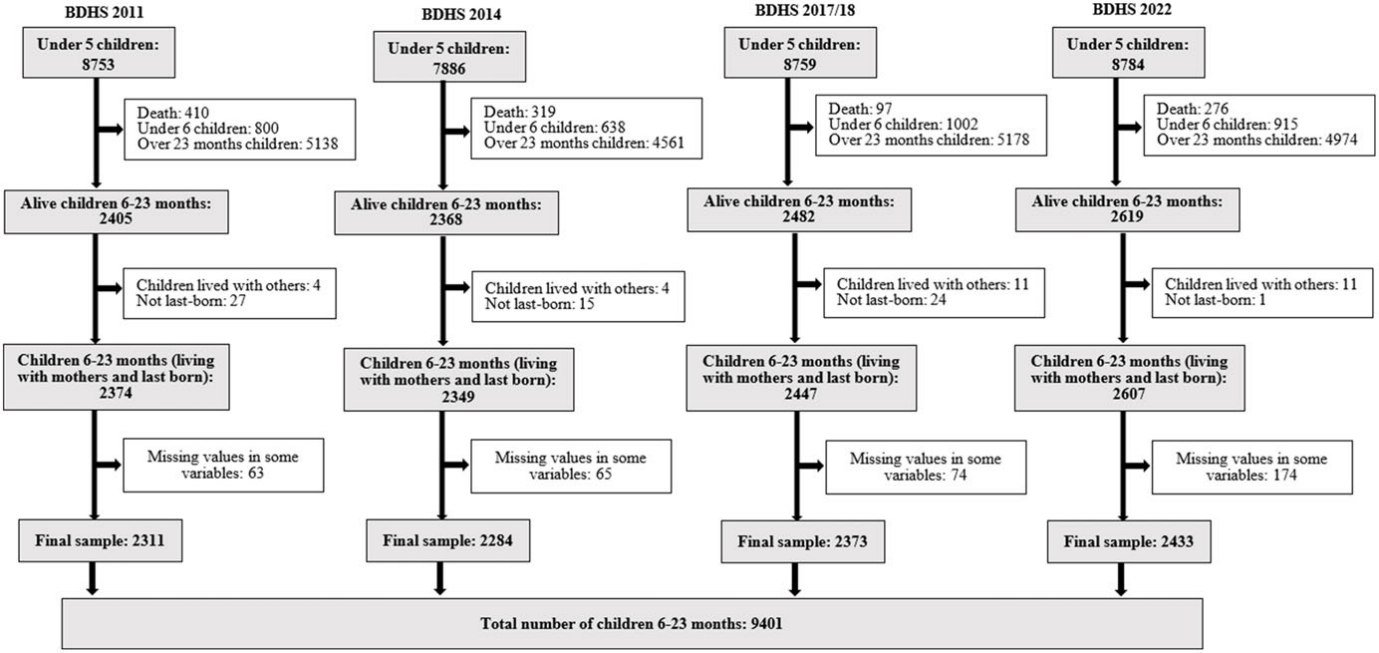
Diagram of sample selection procedure.

The present study utilized data from four consecutive nationally represented Bangladesh Demographic and Health Surveys (BDHS) conducted in 2011, 2014, 2017/18, and 2022. The BDHS employed a cross-sectional two-stage stratified cluster sampling method for data collection, involving face-to-face interviews at households. Initially, enumeration areas or clusters representing a village or group of villages were selected using a probability proportional to size sampling strategy. Then, households from each cluster were selected using an equal probability systematic sampling technique. A detailed description of the methodology of the BDHS can be found elsewhere.^(18,21,26,27)^ The child dataset of BDHS comprised information on children under 5 years of age. In our study, 9401 alive children aged 6–23 months living with mothers and last born were included (2311 in 2011, 2284 in 2014, 2373 in 2017/18, and 2433 in 2022) (Fig. 1).

### Variables of the study

#### Outcome variable

In our study, the consumption of ASF was the outcome variable. In BDHS, mothers/caretakers of children were asked about what types of foods the children had eaten (the child-feeding practice) in the preceding 24-hour period of the survey. For this study, milk, dairy products (yogurt, cheese, or other milk products), fresh/dried fish or shellfish, any meat products (beef, pork, lamb, goat, chicken, or duck), and eggs, were considered as ASF.^(24)^ This outcome variable was categorized into ‘No ASF consumption’ and ‘ASF consumption’.

#### Explanatory variables

In this study, variables having potential influence on ASF consumption were selected based on their availability in BDHS dataset^(18,21,26,27)^ and reported in earlier studies,^(20,23–25,28,29)^ including child characteristics such as, child age in months (6–8, 9–11, 12–17, 18–23), sex, birth order (first, 2–3, ≥4), minimum dietary diversity (no, yes), recent morbidity (no, yes); parental characteristics such as maternal age in years (14–19, 20–29, 20–39, 40–49), religion (Muslim, non-Muslim), maternal education (no formal education, formal education), paternal education (no formal education, formal education), maternal occupation (unemployed, agricultural, non-agricultural), paternal occupation (unemployed, agricultural, non-agricultural), maternal pregnancy status, frequency of antenatal care (ANC) visits (no, 1–3 times, ≥4 times), maternal age of first birth (<20 years, ≥20 years), attitudes toward wife beaten (not justified, justified), maternal access to media (no, yes), family size (≤4 members, ≥5 members); community characteristics such as place of residence (urban, rural), division (Barisal, Chattogram, Dhaka, Khulna, Rajshahi, Rangpur, Sylhet).

Children achieved minimum dietary diversity (MDD) when they consumed food from a minimum of 5 out of the 8 specified food groups (breast milk, grains/roots/tubers/plantains, beans/peas/lentils/nuts/seeds, dairy items, meat/fish/poultry/organ meat, eggs, vitamin A-rich fruits and vegetables, other fruits and vegetables) during the preceding day of the survey.^(30)^ Furthermore, child morbidity was defined as the occurrence of diarrhoea, fever, or cough within the two weeks prior to the survey.^(18)^ Parental occupation was classified according to whether the parent had been employed in the past 12 months. Employed parents were further categorized into agricultural and non-agricultural sectors. Non-agricultural occupations encompassed professional, technical, managerial positions, clerical work, sales and services, skilled and unskilled manual labour, and domestic services.^(31)^ Wife-beating was justified if the mother was beaten by her partner when she burned the food/argued with him/went out without telling him/neglected the children/refused to have sex with him.^(18)^ Maternal media access was defined as having exposure to radio, television, newspapers, or magazines at least once per week.^(18)^ Furthermore, factor analysis of household assets was conducted to construct a wealth index which was further categorized into poorest, poorer, middle, richer, and richest.^(18,21,26,27)^

### Statistical analysis

Since this study utilized the combined dataset of four consecutive BDHS (2011–2022), new strata and cluster variables were constructed to ensure the uniqueness of each BDHS data.^(24)^ To assess the trends of ASF consumption across various explanatory variables, we performed the Cochran–Armitage test.^(32,33)^ In the pooled dataset, a new variable ‘Year of survey’ was created to examine the trends between the survey period. The hierarchical structure of the BDHS dataset, where children were nested within clusters, necessitated the use of multilevel modelling. To account for the clustering effect, a two-stage multilevel mixed-effects logistic regression was conducted to identify both individual- and community-level factors associated with ASF consumption.^(34)^ The analysis was performed separately for each survey year as well as on the pooled dataset from 2011 to 2022. Only potential explanatory variables having P ≤ 0.25 in the bivariable multilevel logistic model were adjusted in the multivariable multilevel logistic regression model.^(35)^ The MDD encompassed the groups considered for ASF consumption in this study. Consequently, in the regression model, we omitted MDD as an explanatory variable and solely illustrated the trends of ASF among the MDD groups. A total of four models were fitted to estimate both the fixed effects of individual- and community-level factors and the random intercept for between-cluster variation. These models included: Model I (Null model without explanatory variables to assess between-cluster variance), Model II (only individual-level factors), Model III (only community-level factors), and Model IV (full model combining both individual- and community-level factors).

The random effects were assessed using the intra-class correlation coefficient (ICC), median odds ratio (MOR), and proportional change in variance (PCV). The ICC quantified the proportion of total variance in ASF consumption attributable to differences between clusters.^(36)^ The MOR, a measure of heterogeneity, represented the median odds ratio for ASF consumption between the cluster with the highest risk and the cluster with the lowest risk when two clusters were randomly chosen. It can be calculated using the following equation: 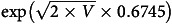 , where V is the between-cluster variance.^(37)^ The PCV indicated the percentage of variance reduction in each subsequent model compared to the null model. It can be calculated using following equation: 
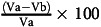
 , where Va is the variance of the null model, and Vb is the variance of each subsequent model.^(36)^ Model fit was evaluated using the log-likelihood, Akaike’s information criterion (AIC), and Bayesian information criterion (BIC), with the model having the lowest AIC and BIC values considered the best fit.^(38)^ The findings of the regression model were presented as crude odds ratios (COR) and adjusted odds ratios (AOR) along with their 95% CI. Exact p-values were reported for all analyses. We checked for multicollinearity using the variance inflation factor (VIF), with a threshold of 10 (Supplementary Table S1). Furthermore, we conducted dominance analysis for rankings of explanatory variables in predicting ASF consumption where the standardized dominance statistics were presented. ‘SVY’ command was used to adjust sampling weight, strata, and cluster while melogit for the regression model. All analyses were conducted in STATA (Version 15.0).

### Ethics statement

This study utilized secondary data from the Demographic and Health Survey (DHS). To access these data, a formal request, detailing the research objectives and purpose, was submitted to the Data Archivist of the DHS Program. Permission to access and use the BDHS datasets was granted for this research. Full access to the data was obtained from www.dhsprogram.com, in compliance with the data sharing policy. The survey authority obtained ethical approval from the Government of Bangladesh, and informed consent was secured from all respondents prior to their participation in the interviews.

## Results

### Background characteristics

A total of 9401 children aged 6–23 months from four consecutive BDHS (2011, 2014, 2017/18, and 2022) were included in this analysis. The majority of the children were aged 12 to 23 months, and around 51% were male. Over the years, dietary diversity among children improved, with the proportion meeting the MDD increasing from 23.8% in 2011 to 37.0% in 2022. However, recent morbidity remained common, with around half of the children experiencing illness across all survey years. In all surveys, most of the mothers were young adults aged 20–29 years, and over 90% of households practised the Muslim religion. Educational attainment of both parents improved, with 94.7% of mothers and 84.8% of fathers having formal education in 2022 compared to 83.1% and 73% in 2011, respectively. Maternal unemployment decreased from 92.6% in 2011 to 73.1% in 2022, with a rise in agricultural and non-agricultural employment. In the case of paternal occupation, there was an increase in non-agricultural activities during this period. Furthermore, the proportion of women receiving four or more ANC visits rose from 26.9% in 2011 to 40.5% in 2022. Maternal early birth (<20 years) and the justification of wife-beating showed a declining trend. Furthermore, half of the mothers had access to media across the surveys. Across all surveys, most participants resided in rural areas, with the majority coming from the Chattogram and Dhaka divisions (Table 1).

**Table 1.** Background characteristics of the participants across the survey years

Characteristics	BDHS 2011	BDHS 2014	BDHS 2017/18	BDHS 2022	Pooled data
Child age in months					
6–8	399 (17.8)	393 (16.2)	403 (16.7)	460 (18.6)	1655 (17.4)
9–11	437 (18.4)	401 (18.4)	387 (16.8)	472 (19.7)	1697 (18.3)
12–17	788 (34.7)	785 (34.2)	824 (34.4)	696 (29.0)	3093 (33.0)
18–23	687 (29.1)	705 (31.2)	759 (32.1)	805 (32.7)	2956 (31.3)
Sex of child					
Male	1175 (50.9)	1172 (52.6)	1229 (51.9)	1226 (49.9)	4802 (51.3)
Female	1136 (49.1)	1112 (47.4)	1144 (48.1)	1207 (50.1)	4599 (48.7)
Birth order					
First	858 (35.6)	940 (39.7)	881 (36.8)	911 (38.7)	3590 (37.7)
2–3	1061 (47.3)	1047 (47.5)	1206 (51.0)	1296 (52.0)	4610 (49.5)
≥4	392 (17.1)	297 (12.8)	286 (12.2)	226 (9.3)	1201 (12.8)
Minimum dietary diversity					
No	1745 (76.2)	1642 (73.4)	1446 (61.5)	1518 (63.0)	6351 (68.4)
Yes	566 (23.8)	642 (26.6)	927 (38.5)	915 (37.0)	3050 (31.6)
Recent morbidity					
No	1046 (44.8)	1100 (47.7)	1019 (43.3)	1233 (51.2)	4398 (46.8)
Yes	1265 (55.2)	1184 (52.3)	1354 (56.7)	1200 (48.8)	5003 (53.2)
Maternal age in years					
14–19	496 (21.0)	513 (22.0)	434 (19.1)	354 (15.2)	1797 (19.3)
20–29	1408 (62.0)	1371 (60.4)	1451 (60.0)	1459 (59.7)	5689 (60.5)
30–39	379 (15.7)	380 (16.6)	465 (20.0)	589 (23.9)	1813 (19.1)
40–49	28 (1.3)	20 (1.0)	23 (0.9)	31 (1.2)	102 (1.1)
Religion					
Muslim	2067 (91.0)	2107 (92.0)	2196 (93.0)	2231 (92.7)	8601 (92.2)
Non-Muslim	244 (9.0)	177 (8.0)	177 (7.0)	202 (7.3)	800 (7.8)
Maternal education					
No formal education	373 (16.9)	284 (13.5)	141 (6.2)	120 (5.3)	918 (10.4)
Formal education	1938 (83.1)	2000 (86.5)	2232 (93.8)	2313 (94.7)	8483 (89.6)
Paternal education					
No formal education	591 (27.0)	493 (23.2)	323 (13.6)	363 (15.2)	1770 (19.6)
Formal education	1720 (73.0)	1791 (76.8)	2050 (86.4)	2070 (84.8)	7631 (80.4)
Maternal occupation					
Unemployed	2130 (92.6)	1774 (75.9)	1457 (61.5)	1820 (73.1)	7181 (75.6)
Agricultural	8 (0.4)	233 (11.7)	652 (27.5)	367 (15.8)	1260 (14.0)
Non-agricultural	173 (7.0)	277 (12.4)	264 (11.0)	246 (11.1)	960 (10.4)
Paternal occupation					
Unemployed	29 (0.9)	15 (0.7)	15 (0.6)	52 (2.1)	111 (1.1)
Agricultural	564 (26.6)	519 (25.0)	451 (19.3)	465 (19.2)	1999 (22.5)
Non-agricultural	1718 (72.5)	1750 (74.3)	1907 (80.1)	1916 (78.7)	7291 (76.4)
Maternal pregnancy status					
No	2224 (96.2)	2213 (97.1)	2305 (97.5)	2359 (97.1)	9101 (97.0)
Yes	87 (3.8)	71 (2.9)	68 (2.5)	74 (2.9)	300 (3.0)
Frequency of ANC					
No	697 (31.9)	466 (21.2)	188 (7.9)	194 (7.5)	1545 (16.9)
1–3 times	933 (41.2)	1084 (48.0)	1055 (45.6)	1227 (52.0)	4299 (46.8)
≥4 times	681 (26.9)	734 (30.8)	1130 (46.5)	1012 (40.5)	3557 (36.3)
Maternal age of first birth					
<20 years	1714 (75.6)	1656 (72.5)	1633 (70.0)	1499 (62.9)	6502 (70.1)
≥20 years	597 (24.4)	628 (27.5)	740 (30.0)	934 (37.1)	2899 (29.9)
Attitudes toward wife beaten					
Not justified	1545 (66.7)	1642 (72.9)	1948 (81.8)	2139 (87.1)	7274 (77.3)
Justified	766 (33.3)	642 (27.1)	425 (18.2)	294 (12.9)	2127 (22.7)
Maternal access to media					
No	1110 (49.8)	1089 (48.9)	1076 (44.0)	1298 (53.7)	4573 (49.1)
Yes	1201 (50.2)	1195 (51.1)	1297 (56.0)	1135 (46.3)	4828 (50.9)
Family size					
≤4 members	667 (29.3)	700 (31.6)	718 (31.0)	752 (32.5)	2837 (31.1)
≥5 members	1644 (70.8)	1584 (68.4)	1655 (69.0)	1681 (67.5)	6564 (68.9)
Wealth index					
Poorest	491 (22.4)	480 (21.9)	504 (20.6)	515 (21.1)	1990 (21.5)
Poorer	441 (20.3)	424 (19.1)	503 (21.6)	501 (21.2)	1869 (20.5)
Middle	439 (19.5)	459 (20.1)	415 (18.4)	475 (20.5)	1788 (19.6)
Richer	471 (20.2)	490 (20.3)	490 (20.4)	478 (19.7)	1929 (20.1)
Richest	469 (17.5)	431 (18.7)	461 (19.1)	464 (17.6)	1825 (18.2)
Place of residence					
Urban	717 (22.8)	733 (26.0)	790 (26.0)	785 (26.2)	3025 (25.2)
Rural	1594 (77.2)	1551 (74.0)	1583 (74.0)	1648 (73.8)	6376 (74.8)
Division					
Barisal	261 (5.6)	267 (5.6)	257 (5.8)	257 (6.0)	1042 (5.7)
Chattogram	478 (23.9)	432 (21.6)	380 (20.4)	408 (21.8)	1698 (21.9)
Dhaka	374 (30.3)	427 (37.5)	634 (34.1)	662 (32.9)	2097 (33.7)
Khulna	263 (9.0)	268 (7.8)	233 (8.8)	273 (10.3)	1037 (9.1)
Rajshahi	295 (13.3)	285 (10.2)	252 (11.6)	241 (10.3)	1073 (11.3)
Rangpur	293 (10.6)	275 (8.9)	280 (11.4)	300 (12.0)	1148 (10.7)
Sylhet	347 (7.3)	330 (8.4)	337 (7.9)	292 (6.7)	1306 (7.6)

*Note:* Number within parenthesis presents percentage.

### Trends in consumption of ASF

**Fig. 2.**
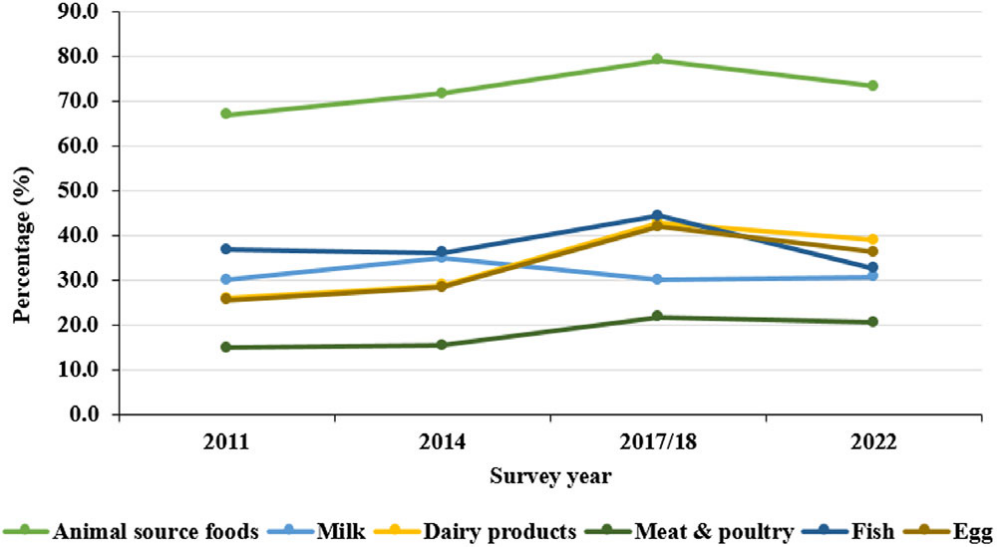
Trends in ASF consumption among children aged 6 to 23 months in Bangladesh, 2011–2022. *Note:* The coloured dots present the percentage and the lines present the trends.

Significant changes in the consumption of various types of ASF were noted among children in Bangladesh from 2011 to 2022. Overall, the intake of ASF increased from 67% in 2011 to 71.6% in 2014, peaking at 79.1% in 2017/18 before declining to 73.3% in 2022 (P < 0.001). Consumption of dairy products rose steadily from 25.9% in 2011 to 28.7% in 2014, reaching 42.8% in 2017/18, but decreased to 38.9% in 2022 (P < 0.001). Similarly, meat and poultry intake increased from 14.8% in 2011 to 15.4% in 2014, peaked at 21.7% in 2017/18, and then slightly dropped to 20.5% in 2022 (P < 0.001). Fish consumption fluctuated, declining from 36.7% in 2011 to 36.1% in 2014, peaking at 44.3% in 2017/18, and then falling to 32.5% in 2022 (P < 0.001). Egg consumption rose from 25.5% in 2011 to 28.4% in 2014, peaked at 41.9% in 2017/18, then decreased to 36.2% in 2022 (P < 0.001). Milk intake, however, remained relatively stable, fluctuating around 30% over the years (P = 0.945) (Fig. 2).

Between 2011 and 2022, the overall consumption of ASF significantly increased among children, regardless of factors such as sex, maternal media exposure, attitudes toward domestic violence, and family size. This upward trend was especially pronounced among younger children aged 6 to 8 months (P < 0.001), 9–11 months (P = 0.012), and 12–17 months (P = 0.016). Children born second or later showed significant increases in ASF consumption, particularly those with birth orders of 2–3 (P = 0.010) and 4 or more (P = 0.001). In addition, children experiencing illness also found a rise in ASF consumption (P < 0.001), as did those from Muslim households (P < 0.001), children of mothers aged 20–29 years (P = 0.001), and 30–39 years (P = 0.007), and those with parents who had received formal education (maternal, P = 0.033; paternal, P = 0.014). Notably, an increase in ASF consumption was observed among children with unemployed mothers (P < 0.001), those whose fathers worked in agriculture (P = 0.047) or non-agricultural occupations (P < 0.001), and children whose mothers were not pregnant (P < 0.001). This trend was particularly significant among children whose mothers had their first child before the age of 20 (P < 0.001), those from both low-income (P < 0.001) and wealthiest households (P = 0.008), and those living in rural areas (P < 0.001) and Dhaka (P < 0.001). ASF consumption consistently increased in these demographics from 2011 to 2014 and again in 2017/18, but it declined in 2022 (Table 2).

**Table 2.** Trends in ASF consumption across the background characteristics among children aged 6 to 23 months in Bangladesh, 2011–2022

Characteristics	BDHS 2011	BDHS 2014	% point change (2011 to 2014)	BDHS 2017/18	% point change (2014 to 2017/18)	BDHS 2022	% point change (2017/18 to 2022)	Overall % point change (2011 to 2022)	P-trends^a^
Child age in months									
6–8	43.3	47.8	4.5	58.1	10.3	53.1	–5.0	9.8	<0.001
9–11	60.2	65.0	4.8	73.6	8.6	68.4	–5.2	8.2	0.012
12–17	71.4	74.6	3.2	82.8	8.2	78.3	–4.5	6.9	0.016
18–23	80.3	84.7	4.4	88.9	4.2	83.2	–5.7	2.9	0.392
Sex of child									
Male	67.9	72.0	4.1	78.7	6.7	74.5	–4.2	6.6	<0.001
Female	66.0	71.2	5.2	79.5	8.3	72.0	–7.5	6.0	0.009
Birth order									
First	74.5	75.2	0.7	82.9	7.7	75.3	–7.6	0.8	0.214
2–3	66.5	70.7	4.2	78.6	7.9	73.2	–5.4	6.7	0.010
≥4	52.5	64.2	11.7	69.6	5.4	65.0	–4.6	12.5	0.001
Minimum dietary diversity									
No	56.6	61.6	5.0	66.7	5.1	58.5	–8.2	1.9	0.714
Yes	100.0	99.3	–0.7	98.9	–0.4	98.3	–0.6	–1.7	0.001
Recent morbidity									
No	71.1	75.9	4.8	81.0	5.1	75.2	–5.8	4.1	0.063
Yes	63.6	67.7	4.1	77.7	10.0	71.2	–6.5	7.6	<0.001
Maternal age in years									
14–19	70.0	73.2	3.2	79.8	6.6	70.1	–9.7	0.1	0.298
20–29	67.4	72.0	4.6	79.9	7.9	73.7	–6.2	6.3	0.001
30–39	63.2	68.5	5.3	76.1	7.6	75.1	–1.0	11.9	0.007
40–49	43.7	67.1	23.4	77.9	10.8	55.5	–22.4	11.8	0.529
Religion									
Muslim	66.3	72.3	6.0	79.1	6.8	73.5	–5.6	7.2	<0.001
Non-Muslim	73.6	63.7	–9.9	78.6	14.9	70.7	–7.9	–2.9	0.876
Maternal education									
No formal education	50.1	54.8	4.7	55.6	0.8	57.4	1.8	7.3	0.174
Formal education	70.3	74.3	4.0	80.7	6.4	74.1	–6.6	3.8	0.033
Paternal education									
No formal education	54.6	65.1	10.5	71.5	6.4	59.3	–12.2	4.7	0.115
Formal education	71.5	73.6	2.1	80.3	6.7	75.8	–4.5	4.3	0.014
Maternal occupation									
Unemployed	66.2	70.4	4.2	78.9	8.5	72.2	–6.7	6.0	<0.001
Agricultural	87.4	74.6	–12.8	77.8	3.2	73.2	–4.6	–14.2	0.690
Non-agricultural	75.1	76.7	1.6	83.4	6.7	80.4	–3.0	5.3	0.100
Paternal occupation									
Unemployed	61.3	64.6	3.3	84.9	20.3	64.6	–20.3	3.3	0.735
Agricultural	63.6	65.1	1.5	74.9	9.8	67.6	–7.3	4.0	0.047
Non-agricultural	68.2	73.9	5.7	80.1	6.2	74.9	–5.2	6.7	<0.001
Maternal pregnancy status									
No	66.3	71.2	4.9	78.7	7.5	73.0	–5.7	6.7	<0.001
Yes	83.9	85.7	1.8	95.8	10.1	83.1	–12.7	–0.8	0.598
Frequency of ANC									
No	53.7	60.7	7.0	69.5	8.8	58.2	–11.3	4.5	0.093
1–3 times	70.8	74.2	3.4	75.7	1.5	71.6	–4.1	0.8	0.597
≥4 times	76.6	75.2	–1.4	84.0	8.8	78.1	–5.9	1.5	0.205
Maternal age of first birth									
<20 years	65.1	71.1	6.0	78.1	7.0	72.2	–5.9	7.1	<0.001
≥20 years	72.6	73.1	0.5	81.5	8.4	75.1	–6.4	2.5	0.542
Attitudes toward wife beaten									
Not justified	67.7	73.9	6.2	79.5	5.6	73.2	–6.3	5.5	0.01
Justified	65.5	65.5	0.0	77.2	11.7	73.6	–3.6	8.1	0.002
Maternal access to media									
No	58.0	64.9	6.9	74.6	9.7	68.6	–6.0	10.6	<0.001
Yes	75.7	78.1	2.4	82.6	4.5	78.6	–4.0	2.9	0.021
Family size									
≤4 members	66.5	74.3	7.8	83.1	8.8	72.1	–11.0	5.6	0.013
≥5 members	67.1	70.4	3.3	77.3	6.9	73.8	–3.5	6.7	<0.001
Wealth index									
Poorest	48.8	60.4	11.6	71.9	11.5	63.5	–8.4	14.7	<0.001
Poorer	61.3	65.3	4.0	76.6	11.3	68.5	–8.1	7.2	0.02
Middle	73.4	74.7	1.3	81.4	6.7	75.9	–5.5	2.5	0.331
Richer	77.3	80.0	2.7	79.7	–0.3	75.5	–4.2	–1.8	0.991
Richest	77.4	78.8	1.4	86.8	8.0	85.0	–1.8	7.6	0.008
Place of residence									
Urban	74.1	76.9	2.8	81.5	4.6	77.2	–4.3	3.1	0.248
Rural	64.8	69.8	5.0	78.3	8.5	71.9	–6.4	7.1	<0.001
Division									
Barisal	63.0	75.5	12.5	76.0	0.5	70.9	–5.1	7.9	0.234
Chattogram	63.9	64.8	0.9	75.3	10.5	63.2	–12.1	–0.7	0.887
Dhaka	64.5	73.5	9.0	81.0	7.5	79.3	–1.7	14.8	<0.001
Khulna	79.1	75.0	–4.1	82.4	7.4	83.4	1.0	4.3	0.059
Rajshahi	76.4	73.8	–2.6	80.9	7.1	82.3	1.4	5.9	0.073
Rangpur	72.0	79.3	7.3	84.2	4.9	70.7	–13.5	–1.3	0.617
Sylhet	50.5	64.5	14.0	69.4	4.9	53.5	–15.9	3.0	0.364

‘–’ indicated decrease.

a The P-value derived from Cochran–Armitage test.

### Predictors of ASF consumption (fixed effects)

In the bivariable multilevel mixed-effect logistic regression analysis, eighteen variables met the criteria (P ≤ 0.25) to be included in the multivariable analysis. These included child age, birth order, morbidity; maternal age, education, occupation, pregnancy status, ANC visit, access to media, age of first birth, attitude towards wife beaten; paternal education, occupation; family size, wealth index, place of residence, division, and year of survey.

A higher odd of ASF consumption was found among children aged 9 to 11 months (AOR = 2.12, 95% CI = 1.75–2.56, P < 0.001), 12 to 17 months (AOR = 3.65, 95% CI = 3.07–4.34, P < 0.001), and 18 to 23 months (AOR = 6.51, 95% CI = 5.38–7.89, P < 0.001) compared to those who were 6 to 8 months of age. Children who had recently experienced illness were 24% less likely to consume ASF (AOR = 0.76, 95% CI = 0.68–0.86, P < 0.001) than those who had not. ASF consumption was about 1.6 times (AOR = 1.60, 95% CI = 1.30–1.98, P < 0.001) higher among children whose mothers had any formal education. Furthermore, maternal involvement in agricultural (AOR = 1.27, 95% CI = 1.04–1.54, P = 0.019) and non-agricultural activities (AOR = 1.36, 95% CI = 1.07–1.72, P = 0.011) were associated with a higher likelihood of consuming ASF among children compared to those whose mothers were unemployed. Maternal pregnancy was associated with 2.5 times (AOR = 2.54, 95% CI = 1.66–3.87, P < 0.001) higher odds of children consuming ASF than those having non-pregnant mothers. Children whose mothers received 1–3 or ≥4 times ANC visits were 1.4 times (AOR = 1.43, 95% CI = 1.20–1.72, P < 0.001) or 1.6 times (AOR = 1.59, 95% CI = 1.29–1.95, P < 0.001) more likely to consume ASF, respectively, compared to those whose mothers had no ANC visits. Moreover, children whose mothers were exposed to any media had a 21% (AOR = 1.21, 95% CI = 1.04–1.39, P = 0.011) higher chance of consuming ASF than those whose mothers were not exposed to any media. Children living in wealthier families, including middle (AOR = 1.82, 95% CI = 1.45–2.30, P < 0.001), richer (AOR = 1.92, 95% CI = 1.52–2.43, P < 0.001), richest (AOR = 2.35, 95% CI = 1.77–3.14, P < 0.001) were more likely to consume ASF compared to those who lived in the poorest families. ASF consumption was found to be lower among children living in Chattogram (AOR = 0.71, 95% CI = 0.57–0.89, P = 0.003) and Sylhet (AOR = 0.53, 95% CI = 0.42–0.69, P < 0.001) while higher those who lived in Khulna (AOR = 1.37, 95% CI = 1.06–1.77, P = 0.017), Rajshahi (AOR = 1.42, 95% CI = 1.09–1.86, P = 0.011) and Rangpur (AOR = 1.36, 95% CI = 1.06–1.75, P = 0.017) compared to Barisal. Furthermore, there were 51% higher odds (AOR = 1.51, 95% CI = 1.25–1.83, P < 0.001) of consuming ASF among children during 2017/18 compared to 2011 (Table 3).

**Table 3.** Factors associated with the consumption of ASF among children 6–23 months of age in Bangladesh (Pooled data)

Characteristics	Unadjusted model		Model I	Model II		Model III		Model IV	
	COR (95% CI)	P-value		AOR (95% CI)	P-value	AOR (95% CI)	P-value	AOR (95% CI)	P-value
Child age in months									
6–8	Ref.			Ref.				Ref.	
9–11	2.10 (1.75, 2.53)	<0.001		2.10 (1.74, 2.54)	<0.001			2.12 (1.75, 2.56)	<0.001
12–17	3.69 (3.11, 4.38)	<0.001		3.64 (3.06, 4.33)	<0.001			3.65 (3.07, 4.34)	<0.001
18–23	6.32 (5.26, 7.59)	<0.001		6.50 (5.37, 7.87)	<0.001			6.51 (5.38, 7.89)	<0.001
Birth order									
First	Ref.			Ref.				Ref.	
2–3	0.79 (0.70, 0.90)	<0.001		0.85 (0.71, 1.01)	0.06			0.86 (0.72, 1.02)	0.092
≥4	0.50 (0.41, 0.60)	<0.001		0.76 (0.57, 1.01)	0.059			0.83 (0.62, 1.10)	0.192
Recent morbidity									
No	Ref.			Ref.				Ref.	
Yes	0.72 (0.64, 0.81)	<0.001		0.76 (0.68, 0.86)	<0.001			0.76 (0.68, 0.86)	<0.001
Maternal age in years									
14–19	Ref.			Ref.				Ref.	
20–29	1.03 (0.88, 1.20)	0.712		1.00 (0.82, 1.23)	0.976			1.01 (0.83, 1.24)	0.895
30–39	0.91 (0.73, 1.13)	0.397		0.99 (0.73, 1.34)	0.93			0.96 (0.71, 1.30)	0.794
40–49	0.51 (0.30, 0.89)	0.018		0.73 (0.40, 1.35)	0.32			0.70 (0.38, 1.29)	0.258
Maternal education									
No formal education	Ref.			Ref.				Ref.	
Formal education	2.61 (2.17, 3.14)	<0.001		1.63 (1.32, 2.01)	<0.001			1.60 (1.30, 1.98)	<0.001
Paternal education									
No formal education	Ref.			Ref.				Ref.	
Formal education	1.93 (1.67, 2.24)	<0.001		1.20 (1.00, 1.44)	0.053			1.15 (0.96, 1.39)	0.126
Maternal occupation									
Unemployed	Ref.			Ref.				Ref.	
Agricultural	1.24 (1.04, 1.47)	0.016		1.49 (1.23, 1.80)	<0.001			1.27 (1.04, 1.54)	0.019
Non-agricultural	1.50 (1.21, 1.86)	<0.001		1.45 (1.14, 1.83)	0.002			1.36 (1.07, 1.72)	0.011
Paternal occupation									
Unemployed	Ref.			Ref.				Ref.	
Agricultural	1.06 (0.63, 1.80)	0.816		1.69 (0.95, 3.01)	0.072			1.43 (0.81, 2.54)	0.218
Non-agricultural	1.45 (0.86, 2.44)	0.162		1.76 (1.00, 3.10)	0.05			1.60 (0.91, 2.81)	0.102
Maternal pregnancy status									
No	Ref.			Ref.				Ref.	
Yes	2.97 (2.00, 4.42)	<0.001		2.30 (1.51, 3.49)	<0.001			2.54 (1.66, 3.87)	<0.001
Frequency of ANC								Ref.	
No	Ref.			Ref.					
1–3 times	1.95 (1.65, 2.30)	<0.001		1.57 (1.32, 1.88)	<0.001			1.43 (1.20, 1.72)	<0.001
≥4 times	2.67 (2.24, 3.19)	<0.001		1.84 (1.51, 2.24)	<0.001			1.59 (1.29, 1.95)	<0.001
Maternal age of first birth									
<20 years	Ref.			Ref.				Ref.	
≥20 years	1.24 (1.07, 1.43)	0.005		0.99 (0.83, 1.18)	0.919			1.04 (0.87, 1.24)	0.671
Attitudes toward wife beaten									
Not justified	Ref.			Ref.				Ref.	
Justified	0.79 (0.68, 0.92)	0.002		0.93 (0.79, 1.10)	0.399			0.94 (0.80, 1.11)	0.468
Maternal access to media									
No	Ref.			Ref.				Ref.	
Yes	1.86 (1.65, 2.09)	<0.001		1.29 (1.12, 1.48)	0.001			1.21 (1.04, 1.39)	0.011
Family size									
≤4 members	Ref.			Ref.				Ref.	
≥5 members	0.91 (0.81, 1.04)	0.159		0.99 (0.87, 1.14)	0.941			1.03 (0.90, 1.19)	0.643
Wealth index									
Poorest	Ref.			Ref.				Ref.	
Poorer	1.37 (1.14, 1.64)	0.001		1.15 (0.94, 1.40)	0.174			1.16 (0.95, 1.41)	0.156
Middle	2.13 (1.75, 2.60)	<0.001		1.73 (1.37, 2.17)	<0.001			1.82 (1.45, 2.30)	<0.001
Richer	2.43 (2.00, 2.96)	<0.001		1.75 (1.39, 2.20)	<0.001			1.92 (1.52, 2.43)	<0.001
Richest	3.17 (2.55, 3.94)	<0.001		2.04 (1.56, 2.67)	<0.001			2.35 (1.77, 3.14)	<0.001
Place of residence									
Urban	Ref.					Ref.		Ref.	
Rural	0.71 (0.62, 0.82)	<0.001				0.73 (0.64, 0.83)	<0.001	1.13 (0.96, 1.32)	0.135
Division									
Barisal	Ref.					Ref.		Ref.	
Chattogram	0.85 (0.68, 1.06)	0.156				0.83 (0.67, 1.03)	0.096	0.71 (0.57, 0.89)	0.003
Dhaka	1.28 (1.03, 1.60)	0.027				1.18 (0.95, 1.46)	0.141	1.04 (0.83, 1.29)	0.757
Khulna	1.73 (1.34, 2.23)	<0.001				1.70 (1.32, 2.18)	<0.001	1.37 (1.06, 1.77)	0.017
Rajshahi	1.57 (1.21, 2.04)	0.001				1.57 (1.21, 2.03)	0.001	1.42 (1.09, 1.86)	0.011
Rangpur	1.37 (1.07, 1.74)	0.012				1.37 (1.08, 1.74)	0.011	1.36 (1.06, 1.75)	0.017
Sylhet	0.57 (0.45, 0.72)	<0.001				0.57 (0.45, 0.72)	<0.001	0.53 (0.42, 0.69)	<0.001
Survey year									
2011	Ref.					Ref.		Ref.	
2014	1.25 (1.06, 1.48)	0.009				1.26 (1.06, 1.48)	0.007	1.11 (0.93, 1.32)	0.262
2017/18	1.92 (1.62, 2.27)	<0.001				1.90 (1.61, 2.25)	<0.001	1.51 (1.25, 1.83)	<0.001
2022	1.37 (1.16, 1.62)	<0.001				1.35 (1.14, 1.59)	<0.001	1.10 (0.92, 1.32)	0.313
Random effects (measures of variations)									
Cluster level variance (SE)			0.61 (0.07)	0.51 (0.07)		0.46 (0.06)		0.44 (0.07)	
P-value			<0.001	<0.001		<0.001		<0.001	
PCV (%)			Ref.	16.40		24.60		27.90	
ICC (%)			15.70	13.40		12.30		11.70	
MOR			2.10	1.97		1.90		1.88	
Fit criteria (model diagnostics)									
Loglikelihood			–5470.69	–4884.47		–5376.35		–4828.80	
AIC			10945.38	9824.94		10776.70		9733.60	
BIC			10959.68	10025.09		10862.48		10005.23	

COR, crude odds ratio; AOR, adjusted odds ratio; Ref, reference; SE, standard error, PCV, proportional change in variance; ICC, intra-cluster correlation coefficient; MOR, median odds ratio; AIC, Akaike’s information criterion; BIC, Bayesian information criterion.

### Random effects and model fitness

The null model revealed significant variability in the odds of ASF consumption among children across clusters (0.61) (P < 0.001). This significant variation persisted in the subsequent models, suggesting that these factors significantly account for the variability (P < 0.001). Furthermore, the null model showed that 15.7% of the variance in the odds of consuming ASF in Bangladesh can be attributed to differences between clusters. However, in Model IV, which adjusted for individual and community-level factors, the cluster-level variance dropped to 11.7%, indicating that both sets of variables explained much of the between-cluster differences in ASF consumption. In Model IV, the AIC and BIC values were lower compared to other models, and the log-likelihood ratio was the highest, indicating that Model IV provided the best fit for predicting ASF consumption among children. The MOR in Model IV showed that if a child moved from a cluster with a lower likelihood of consuming ASF to a higher likelihood, the median increase in the odds of consuming ASF would be 1.88 times. The final model (Model IV) showed a PCV of 27.9%, suggesting that 27.9% of the variation in ASF consumption was explained by the combined individual- and community-level factors (Table 3).

### Rankings of explanatory variables in predicting ASF consumption

**Fig. 3.**
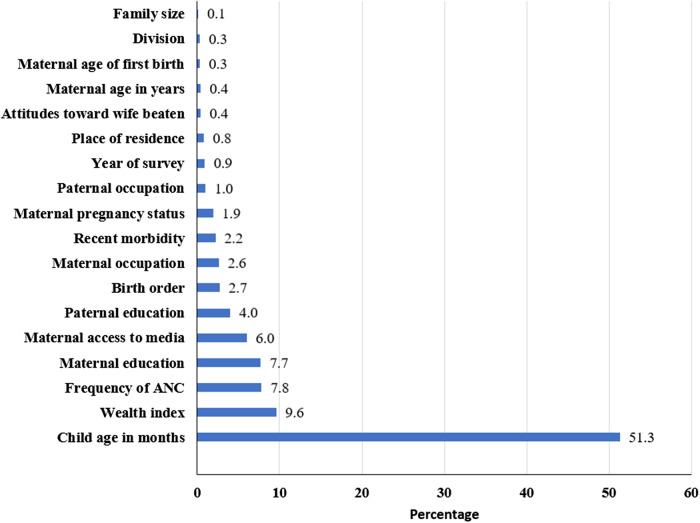
Rankings of explanatory variables in predicting ASF consumption from pooled data.

The standardized dominance statistics suggested that child age was the most influential predictor of ASF consumption. Other influential predictors included the household wealth index, frequency of ANC visits, and maternal education, which ranked second, third, and fourth, respectively. Subsequent rankings revealed the importance of factors such as maternal access to media, paternal education, birth order, maternal occupation, and recent morbidity (Fig. 3).

## Discussion

Dietary intake of ASF is crucial for children’s optimal growth and development. This study investigated the trends and factors influencing ASF consumption among children aged 6–23 months in Bangladesh. The data utilized in this study were derived from four back-to-back BDHS databases. However, to ensure enough statistical power from a large sample size, the results of the pooled dataset were considered. This study found a significant improvement in the consumption of ASF in Bangladesh. It also identified several factors, including child characteristics (child age, recent morbidity), parental characteristics (maternal education, occupation, pregnancy status, frequency of ANC visits, and access to media), wealth index, division, and year of the survey as critical determinants of ASF consumption in Bangladesh.

The present study found that the overall consumption of ASF increased between 2011 to 2017/8. A similar trend of rising consumption of meat/fish/egg (53.0% to 67.2%) among children was observed during this period in Bangladesh.^(29)^ Several factors could contribute to this shift, such as improved household economies, nutrition awareness programmes, increased agricultural productivity, and better food distribution. In Bangladesh, nutritional interventions significantly increased ASF consumption among children under two years of age.^(39)^ Furthermore, the production of ASF in Bangladesh more than doubled between 2012/13 and 2021/22.^(40,41)^ However, our study also revealed a decline in ASF consumption between 2017/18 and 2022. Despite increased ASF production, rising food prices due to inflation may have reduced access. Food inflation rates climbed from 7.62% in October 2017 (the start of BDHS 2017/18 data collection) to 8.37% in June 2022 (the start of BDHS 2022 data collection), likely contributing to this decline.^(42,43)^ Furthermore, economic shocks from the COVID-19 pandemic may have further constrained household purchasing power, limiting consumption of more expensive foods like ASF during this period.^(44)^

Our study found that older children had a higher likelihood of consuming ASF. This finding was concordant with earlier studies in Bangladesh and other South Asian Nations where older children exhibited higher intake across all food groups. It demonstrated more appropriate complementary feeding practices.^(45–47)^ As children grow older, their nutritional needs and feeding abilities evolve, likely contributing to a natural progression towards higher consumption of ASF. Consumption increased as increasing child age would be more than changing through other variables. This suggests that child age was the study’s most influential predictor of ASF consumption.

Acute illnesses in children often harm appetite, resulting in lower consumption and absorption of essential nutrients.^(9,48,49)^ This was also observed in our study, as children with recent illnesses showed reduced intake of ASF. Such findings emphasize that illness potentially contributes to child malnutrition.^(50)^

In line with another study, our findings highlighted the role of maternal education in ASF consumption among young children.^(24)^ Poor maternal education was among the strongest factors of inadequate complementary feeding in South Asian countries.^(46)^ Maternal education increases their empowerment and decision-making powers. It also enhances their access to nutritional knowledge, fosters shifts in societal dietary norms, promotes health-conscious behaviours, and improves access to resources like healthcare facilities. These factors could lead to better complementary feeding practices, such as offering balanced, nutritious meals and integrating ASF to enhance children’s overall health.^(51)^ Thereby, nutrition education intervention has been identified as crucial for promoting ideal feeding practices and mitigating adverse nutritional outcomes among children in Bangladesh.^(52)^

In agreement with earlier studies, maternal employment was associated with a higher ASF consumption among children.^(53)^ Children of working mothers were usually able to get a more diversified diet than children with homemaker mothers.^(53)^ A working mother may have greater control over food choices owing to improved accessibility and affordability. Besides, employed mothers have more significant health and nutritional knowledge, potentially positively influencing feeding practices.^(51)^

This study found a positive association between maternal pregnancy and ASF consumption among children. Pregnancy often influences household dietary patterns.^(54)^ Increased attention to maternal health might result in changes in overall food choices within the household, potentially leading to the availability and consumption of more ASF. In Bangladesh, a study found that approximately 86% of pregnant women consumed flesh foods.^(55)^ Women receiving prenatal care or attending healthcare facilities might receive advice on the importance of a diverse and nutrient-rich diet during pregnancy, including ASF, to meet the nutritional needs of both mother and child. Nutrition counselling has been shown to significantly increase ASF consumption among pregnant women and their children.^(56,57)^ Previous studies indicate maternal dietary choices strongly influence children’s diet quality, particularly ASF in Bangladesh.^(57,58)^

The frequency of ANC visits demonstrated a significant association with ASF consumption in our research, as observed in an earlier study.^(25)^ In another study, higher maternal ANC visits during pregnancy increased child dietary diversity and minimum meal frequency, leading to increased consumption of more ASF in Bangladesh.^(47)^ It was also evident that inadequate ANC was a predictor of inappropriate child-feeding practices in South Asian countries.^(46)^ Furthermore, maternal ANC appointments improved maternal knowledge of health, nutrition, and child-feeding practices. This increase in maternal nutritional knowledge contributed to higher consumption of nutrient-dense foods, including flesh/eggs/nuts/legumes in Bangladesh.^(59)^ Furthermore, mass-media exposure improved child-feeding practices in Bangladesh through advertisements and direct messages which influenced mother to feed their children appropriately.^(46,60)^

Lower consumption of ASF in economically disadvantaged households was also evident in earlier research, which highlighted the unequal distribution of nutrients like protein, fat, and energy across different income groups.^(61)^ A study found that the consumption of ASF was twice as high in non-poor households compared to very poor households.^(62)^ The underlying reason was often attributed to the higher cost of ASF compared to plant-based alternatives. Therefore, poor people did not have the purchasing power to buy these nutrient-rich ASF for their children.^(61)^

In line with earlier study, our study found ASF consumption was higher among individuals who consumed a diversified diet.^(63)^ Dietary diversity is a proxy indicator of diet quality and a good quality diet should incorporate nutrient-rich ASF. Children who achieved MDD were likely to have a more varied and balanced diet, including various food groups. Of the eight food groups comprising MDD criteria, three were ASF. Thus, the inclusion of ASF was anticipated in this diversified diet.^(64)^

Despite the earlier increase, the recent decline in ASF consumption highlights the urgent need for sustained efforts to address economic and social barriers to ASF access. Policymakers should focus on vulnerable populations by addressing socioeconomic disparities and enhancing maternal education, particularly regarding child nutrition and feeding practices. Additionally, increasing employment opportunities for women, expanding access to ANC services, and increasing media exposure for nutrition-related messaging could further boost ASF consumption. Given the lower odds of ASF intake among children in Chattogram and Sylhet, region-specific programmes are essential to address geographic disparities. Policymakers should also prioritize support for lower-income families. Overall, strengthening nutrition policies and social protection programmes, while addressing economic constraints like food inflation, could help reverse the declining trend in ASF consumption and ensure sustained improvements in child nutrition.

This study exhibited notable strengths and limitations that shaped its findings. Its major strength lies in the use of a large representative sample size covering all regions in Bangladesh. This broad coverage, facilitated by a meticulous sampling technique using sampling weights, significantly enhanced the study’s credibility, ensuring precise and comprehensive findings regarding ASF consumption among children aged 6–23 months. Further, the robustness of the data analysis was another strength of this study. However, limitations existed within the study design. Its cross-sectional nature restricted the establishment of direct cause-and-effect relationships between the variables studied. Additionally, the study solely identified the subjective nature of the responses to ASF consumption without specifying quantities or frequency, potentially limiting the depth of insight into dietary patterns.

## Conclusion

The consumption of ASF among children aged 6–23 months improved significantly between 2011 and 2017/18, but a decline was observed in 2022. Various factors influenced ASF consumption, including the child’s age, health status, maternal education, occupation, pregnancy status, frequency of ANC visits, and household wealth index. The considerable number of children lacking ASF in their diets emphasized the need for targeted interventions. To sustain and further advance the improvements, it is essential for the government and policymakers to prioritize these critical areas.

## Supporting information

Hassan et al. supplementary material Hassan et al. supplementary material

## Data Availability

All data are freely available from the DHS: https://www.dhsprogram.com/Countries/Country-Main.cfm?ctry_id=1&c=Bangladesh&Country=Bangladesh&cn=&r=4

## Supplementary material

The supplementary material for this article can be found at https://doi.org/10.1017/jns.2025.7
