# Supplementary material for: Trends and associated factors of animal source foods consumption among children aged 6–23 months in Bangladesh: evidence from four consecutive national surveys
Source: J Nutr Sci. 2025 Feb 21;14:e20. doi: 10.1017/jns.2025.7 (PMC11867821; doi:10.1017/jns.2025.7)
Supplement: Hassan et al. supplementary material [file S2048679025000072sup001.docx]

**Supplementary material**

**Table S1.** Variance inflation (VIF) values of models

| **Characteristics** | **VIF** | | | | |
| --- | --- | --- | --- | --- | --- |
|  | **BDHS 2011** | **BDHS**  **2014** | **BDHS**  **2017/18** | **BDHS**  **2022** | **Pooled**  **data** |
| **Child age in months** | 1.04 | 1.03 | 1.01 | 1.03 | 1.03 |
| **Birth order** | 2.10 | 1.18 | 1.14 | 1.92 | 2.08 |
| **Recent morbidity** | 1.01 | 1.0 | 1.01 | 1.02 | 1.01 |
| **Maternal age in years** | 2.13 | - | - | 2.04 | 2.14 |
| **Religion** | 1.03 | - | - | - | - |
| **Maternal education** | 1.38 | 1.29 | 1.12 | 1.17 | 1.28 |
| **Paternal education** | 1.35 | 1.31 | 1.16 | 1.21 | 1.28 |
| **Maternal occupation** | 1.02 | 1.05 | 1.04 | 1.04 | 1.04 |
| **Paternal occupation** | - | - | - | 1.06 | 1.11 |
| **Maternal pregnancy status** | 1.03 | 1.03 | 1.03 | 1.02 | 1.02 |
| **Frequency of ANC** | 1.32 | 1.25 | 1.22 | 1.18 | 1.30 |
| **Maternal age of first birth** | 1.37 | - | 1.10 | 1.35 | 1.37 |
| **Attitudes toward wife beaten** | 1.04 | 1.06 | - | - | 1.06 |
| **Maternal access to media** | 1.53 | 1.63 | 1.37 | 1.15 | 1.37 |
| **Family size** | - | 1.08 | 1.04 | 1.05 | 1.05 |
| **Wealth index** | 1.97 | 2.02 | 1.81 | 1.49 | 1.81 |
| **Place of residence** | 1.31 | 1.30 | 1.24 | 1.27 | 1.29 |
| **Division** | 1.04 | 1.03 | 1.06 | 1.03 | 1.04 |
| **Year of survey** | - | - | - | - | 1.14 |

**Table S2.** Factors associated with the consumption of ASF among children 6 to 23 months of age in 2011.

| **Characteristics** | **Unadjusted model** | | **Model I** | **Model II** | | **Model III** | | **Model IV** | |
| --- | --- | --- | --- | --- | --- | --- | --- | --- | --- |
|  | **COR [95%CI]** | **P value** |  | **AOR [95%CI]** | **P value** | **AOR [95%CI]** | **P value** | **AOR [95%CI]** | **P value** |
| **Child age in months** |  |  |  |  |  |  |  |  |  |
| 6–8 | Ref. |  |  | Ref. |  |  |  | Ref. |  |
| 9–11 | 2.12 [1.49, 3.04] | <0.001 |  | 2.19 [1.52, 3.15] | <0.001 |  |  | 2.21 [1.53, 3.19] | <0.001 |
| 12–17 | 3.72 [2.70, 5.12] | <0.001 |  | 3.76 [2.68, 5.27] | <0.001 |  |  | 3.75 [2.67, 5.28] | <0.001 |
| 18–23 | 6.22 [4.45, 8.69] | <0.001 |  | 6.89 [4.83, 9.81] | <0.001 |  |  | 6.96 [4.87, 9.94] | <0.001 |
| **Birth order** |  |  |  |  |  |  |  |  |  |
| First | Ref. |  |  | Ref. |  |  |  | Ref. |  |
| 2 to 3 | 0.66 [0.52, 0.84] | 0.001 |  | 0.77 [0.55, 1.07] | 0.124 |  |  | 0.78 [0.56, 1.09] | 0.147 |
| ≥4 | 0.39 [0.28, 0.54] | <0.001 |  | 0.67 [0.40, 1.13] | 0.135 |  |  | 0.73 [0.43, 1.23] | 0.239 |
| **Recent morbidity** |  |  |  |  |  |  |  |  |  |
| No | Ref. |  |  | Ref. |  |  |  | Ref. |  |
| Yes | 0.67 [0.53, 0.83] | <0.001 |  | 0.73 [0.58, 0.93] | 0.009 |  |  | 0.75 [0.59, 0.95] | 0.015 |
| **Maternal age in years** |  |  |  |  |  |  |  |  |  |
| 14-19 | Ref. |  |  | Ref. |  |  |  | Ref. |  |
| 20-29 | 0.90 [0.68, 1.20] | 0.479 |  | 0.95 [0.64, 1.42] | 0.819 |  |  | 0.96 [0.64, 1.44] | 0.842 |
| 30-39 | 0.74 [0.51, 1.05] | 0.095 |  | 0.97 [0.53, 1.78] | 0.930 |  |  | 0.94 [0.51, 1.71] | 0.829 |
| 40-49 | 0.31 [0.10, 0.95] | 0.041 |  | 0.43 [0.13, 1.43] | 0.170 |  |  | 0.40 [0.12, 1.31] | 0.130 |
| **Religion** |  |  |  |  |  |  |  |  |  |
| Muslim | Ref. |  |  | Ref. |  |  |  | Ref. |  |
| Non-Muslim | 1.35 [0.96, 1.90] | 0.080 |  | 1.10 [0.74, 1.63] | 0.650 |  |  | 1.10 [0.74, 1.65] | 0.638 |
| **Maternal education** |  |  |  |  |  |  |  |  |  |
| No formal education | Ref. |  |  | Ref. |  |  |  | Ref. |  |
| Formal education | 2.44 [1.84, 3.24] | <0.001 |  | 1.19 [0.82, 1.72] | 0.352 |  |  | 1.15 [0.79, 1.66] | 0.465 |
| **Paternal education** |  |  |  |  |  |  |  |  |  |
| No formal education | Ref. |  |  | Ref. |  |  |  | Ref. |  |
| Formal education | 2.12 [1.68, 2.68] | <0.001 |  | 1.22 [0.91, 1.64] | 0.180 |  |  | 1.19 [0.89, 1.61] | 0.245 |
| **Maternal occupation** |  |  |  |  |  |  |  |  |  |
| Unemployed | Ref. |  |  | Ref. |  |  |  | Ref. |  |
| Agricultural | 3.72 [0.35, 39.72] | 0.277 |  | 4.96 [0.46, 53.17] | 0.186 |  |  | 4.85 [0.39, 60.91] | 0.222 |
| Non-agricultural | 1.42 [0.92, 2.21] | 0.117 |  | 1.14 [0.68, 1.93] | 0.623 |  |  | 1.12 [0.66, 1.90] | 0.671 |
| **Maternal pregnancy status** |  |  |  |  |  |  |  |  |  |
| No | Ref. |  |  | Ref. |  |  |  | Ref. |  |
| Yes | 3.07 [1.53, 6.16] | 0.002 |  | 2.06 [0.90, 4.69] | 0.087 |  |  | 2.22 [0.96, 5.13] | 0.061 |
| **Frequency of ANC** |  |  |  |  |  |  |  |  |  |
| No | Ref. |  |  | Ref. |  |  |  | Ref. |  |
| 1-3 times | 2.22 [1.71, 2.87] | <0.001 |  | 1.75 [1.33, 2.30] | <0.001 |  |  | 1.58 [1.20, 2.08] | 0.001 |
| ≥4 times | 3.08 [2.28, 4.17] | <0.001 |  | 2.03 [1.43, 2.86] | <0.001 |  |  | 1.79 [1.25, 2.56] | 0.001 |
| **Maternal age of first birth** |  |  |  |  |  |  |  |  |  |
| <20 years | Ref. |  |  | Ref. |  |  |  | Ref. |  |
| ≥20 years | 1.42 [1.10, 1.85] | 0.008 |  | 1.08 [0.77, 1.52] | 0.638 |  |  | 1.18 [0.84, 1.65] | 0.338 |
| **Attitudes toward wife beaten** |  |  |  |  |  |  |  |  |  |
| Not justified | Ref. |  |  | Ref. |  |  |  | Ref. |  |
| Justified | 0.86 [0.68, 1.10] | 0.231 |  | 1.07 [0.82, 1.39] | 0.614 |  |  | 1.05 [0.81, 1.36] | 0.732 |
| **Maternal access to media** |  |  |  |  |  |  |  |  |  |
| No | Ref. |  |  | Ref. |  |  |  | Ref. |  |
| Yes | 2.32 [1.85, 2.90] | <0.001 |  | 1.35 [1.02, 1.78] | 0.033 |  |  | 1.33 [1.00, 1.75] | 0.048 |
| **Wealth index** |  |  |  |  |  |  |  |  |  |
| Poorest | Ref. |  |  | Ref. |  |  |  | Ref. |  |
| Poorer | 1.66 [1.17, 2.37] | 0.005 |  | 1.27 [0.86, 1.89] | 0.233 |  |  | 1.30 [0.87, 1.94] | 0.196 |
| Middle | 3.12 [2.21, 4.40] | <0.001 |  | 2.17 [1.47, 3.20] | <0.001 |  |  | 2.34 [1.58, 3.48] | <0.001 |
| Richer | 4.22 [2.91, 6.11] | <0.001 |  | 2.60 [1.68, 4.03] | <0.001 |  |  | 2.95 [1.88, 4.62] | <0.001 |
| Richest | 4.46 [2.99, 6.67] | <0.001 |  | 2.12 [1.28, 3.48] | 0.003 |  |  | 2.67 [1.58, 4.51] | <0.001 |
| **Place of residence** |  |  |  |  |  |  |  |  |  |
| Urban | Ref. |  |  |  |  | Ref. |  | Ref. |  |
| Rural | 0.61 [0.46, 0.79] | <0.001 |  |  |  | 0.58 [0.45, 0.76] | <0.001 | 1.19 [0.87, 1.62] | 0.282 |
| **Division** |  |  |  |  |  |  |  |  |  |
| Barisal | Ref. |  |  |  |  | Ref. |  | Ref. |  |
| Chattogram | 1.08 [0.73, 1.61] | 0.687 |  |  |  | 1.03 [0.70, 1.52] | 0.882 | 1.10 [0.74, 1.64] | 0.648 |
| Dhaka | 1.13 [0.76, 1.70] | 0.543 |  |  |  | 1.00 [0.67, 1.50] | 0.989 | 1.09 [0.72, 1.66] | 0.669 |
| Khulna | 2.49 [1.52, 4.07] | <0.001 |  |  |  | 2.42 [1.48, 3.94] | <0.001 | 2.02 [1.22, 3.35] | 0.006 |
| Rajshahi | 2.10 [1.30, 3.39] | 0.002 |  |  |  | 2.07 [1.29, 3.33] | 0.003 | 2.12 [1.29, 3.50] | 0.003 |
| Rangpur | 1.58 [1.02, 2.46] | 0.042 |  |  |  | 1.60 [1.03, 2.48] | 0.037 | 1.93 [1.21, 3.08] | 0.006 |
| Sylhet | 0.58 [0.39, 0.88] | 0.010 |  |  |  | 0.59 [0.39, 0.89] | 0.012 | 0.60 [0.38, 0.94] | 0.025 |
| **Random-effects (measures of variations)** |  |  |  |  |  |  |  |  |  |
| Cluster level variance (SE) |  |  | 0.59 (0.13) | 0.53 (0.14) |  | 0.46 (12.00) |  | 0.45 (0.14) |  |
| P‐value |  |  |  | <0.001 |  | <0.001 |  | <0.001 |  |
| PCV (%) |  |  | Ref. | 10.20 |  | 22.30 |  | 23.70 |  |
| ICC (%) |  |  | 15.20 | 13.80 |  | 12.30 |  | 12.10 |  |
| MOR |  |  | 2.08 | 1.99 |  | 1.92 |  | 1.90 |  |
| **Fit criteria (model diagnostics)** |  |  |  |  |  |  |  |  |  |
| Loglikelihood |  |  | -1437.56 | -1246.52 |  | -1408.67 |  | -1228.66 |  |
| AIC |  |  | 2879.12 | 2545.03 |  | 2835.35 |  | 2523.32 |  |
| BIC |  |  | 2890.60 | 2694.37 |  | 2887.04 |  | 2712.86 |  |

COR, crude odds ratio; CI, confidence interval; AOR, adjusted odds ratio; Ref, reference; SE, standard error, PCV, proportional change in variance; ICC, intra-cluster correlation coefficient; MOR, median odds ratio; AIC, Akaike’s information criterion; BIC, Bayesian information criterion.

**Table S3.** Factors associated with the consumption of ASF among children 6 to 23 months of age in 2014.

| **Characteristics** | **Unadjusted model** | | **Model I** | **Model II** | | **Model III** | | **Model IV** | |
| --- | --- | --- | --- | --- | --- | --- | --- | --- | --- |
|  | **COR [95%CI]** | **P value** |  | **AOR [95%CI]** | **P value** | **AOR [95%CI]** | **P value** | **AOR [95%CI]** | **P value** |
| **Child age in months** |  |  |  |  |  |  |  |  |  |
| 6–8 | Ref. |  |  | Ref. |  |  |  | Ref. |  |
| 9–11 | 2.25 [1.51, 3.35] | <0.001 |  | 2.22 [1.46, 3.37] | <0.001 |  |  | 2.24 [1.47, 3.41] | <0.001 |
| 12–17 | 3.61 [2.48, 5.25] | <0.001 |  | 3.77 [2.55, 5.58] | <0.001 |  |  | 3.80 [2.57, 5.62] | <0.001 |
| 18–23 | 7.27 [4.78, 11.06] | <0.001 |  | 7.35 [4.71, 11.46] | <0.001 |  |  | 7.40 [4.74, 11.56] | <0.001 |
| **Birth order** |  |  |  |  |  |  |  |  |  |
| First | Ref. |  |  | Ref. |  |  |  | Ref. |  |
| 2 to 3 | 0.80 [0.61, 1.05] | 0.105 |  | 0.85 [0.62, 1.16] | 0.312 |  |  | 0.86 [0.63, 1.17] | 0.339 |
| ≥4 | 0.55 [0.37, 0.81] | 0.003 |  | 0.77 [0.49, 1.22] | 0.272 |  |  | 0.80 [0.51, 1.26] | 0.342 |
| **Recent morbidity** |  |  |  |  |  |  |  |  |  |
| No | Ref. |  |  | Ref. |  |  |  | Ref. |  |
| Yes | 0.63 [0.48, 0.82] | 0.001 |  | 0.69 [0.53, 0.89] | 0.005 |  |  | 0.70 [0.53, 0.91] | 0.008 |
| **Maternal education** |  |  |  |  |  |  |  |  |  |
| No formal education | Ref. |  |  | Ref. |  |  |  | Ref. |  |
| Formal education | 2.32 [1.64, 3.28] | <0.001 |  | 1.61 [1.10, 2.34] | 0.014 |  |  | 1.64 [1.12, 2.40] | <0.001 |
| **Paternal education** |  |  |  |  |  |  |  |  |  |
| No formal education | Ref. |  |  | Ref. |  |  |  | Ref. |  |
| Formal education | 1.51 [1.07, 2.14] | 0.020 |  | 0.99 [0.65, 1.51] | 0.962 |  |  | 0.97 [0.64, 1.48] | 0.888 |
| **Maternal occupation** |  |  |  |  |  |  |  |  |  |
| Unemployed | Ref. |  |  | Ref. |  |  |  | Ref. |  |
| Agricultural | 1.33 [0.90, 1.95] | 0.147 |  | 1.61 [1.07, 2.42] | 0.022 |  |  | 1.53 [1.01, 2.32] | 0.045 |
| Non-agricultural | 1.42 [0.93, 2.17] | 0.106 |  | 1.47 [0.94, 2.30] | 0.095 |  |  | 1.44 [0.91, 2.26] | 0.116 |
| **Maternal pregnancy status** |  |  |  |  |  |  |  |  |  |
| No | Ref. |  |  | Ref. |  |  |  | Ref. |  |
| Yes | 3.21 [1.31, 7.91] | 0.011 |  | 2.65 [1.12, 6.24] | 0.026 |  |  | 2.99 [1.27, 7.08] | 0.013 |
| **Frequency of ANC** |  |  |  |  |  |  |  |  |  |
| No | Ref. |  |  | Ref. |  |  |  | Ref. |  |
| 1-3 times | 1.74 [1.20, 2.51] | 0.003 |  | 1.55 [1.05, 2.30] | 0.028 |  |  | 1.52 [1.03, 2.23] | 0.035 |
| ≥4 times | 1.76 [1.21, 2.55] | 0.003 |  | 1.31 [0.86, 1.99] | 0.215 |  |  | 1.22 [0.80, 1.85] | 0.351 |
| **Attitudes toward wife beaten** |  |  |  |  |  |  |  |  |  |
| Not justified | Ref. |  |  | Ref. |  |  |  | Ref. |  |
| Justified | 0.63 [0.46, 0.87] | 0.005 |  | 0.74 [0.53, 1.03] | 0.074 |  |  | 0.74 [0.53, 1.03] | 0.075 |
| **Maternal access to media** |  |  |  |  |  |  |  |  |  |
| No | Ref. |  |  | Ref. |  |  |  | Ref. |  |
| Yes | 1.90 [1.49, 2.44] | <0.001 |  | 1.24 [0.91, 1.71] | 0.178 |  |  | 1.22 [0.88, 1.68] | 0.233 |
| **Family size** |  |  |  |  |  |  |  |  |  |
| ≤4 members | Ref. |  |  | Ref. |  |  |  | Ref. |  |
| ≥5 members | 0.78 [0.58, 1.03] | 0.082 |  | 0.87 [0.65, 1.16] | 0.348 |  |  | 0.90 [0.67, 1.20] | 0.464 |
| **Wealth index** |  |  |  |  |  |  |  |  |  |
| Poorest | Ref. |  |  | Ref. |  |  |  | Ref. |  |
| Poorer | 1.24 [0.80, 1.94] | 0.334 |  | 1.15 [0.72, 1.83] | 0.556 |  |  | 1.20 [0.75, 1.92] | 0.441 |
| Middle | 2.04 [1.27, 3.28] | 0.003 |  | 1.85 [1.06, 3.22] | 0.031 |  |  | 1.99 [1.15, 3.47] | 0.015 |
| Richer | 2.81 [1.85, 4.26] | <0.001 |  | 2.23 [1.33, 3.72] | 0.002 |  |  | 2.44 [1.44, 4.14] | 0.001 |
| Richest | 2.56 [1.60, 4.11] | <0.001 |  | 2.06 [1.14, 3.71] | 0.017 |  |  | 2.36 [1.26, 4.44] | 0.007 |
| **Place of residence** |  |  |  |  |  |  |  |  |  |
| Urban | Ref. |  |  |  |  | Ref. |  | Ref. |  |
| Rural | 0.72 [0.55, 0.95] | 0.018 |  |  |  | 0.71 [0.55, 0.93] | 0.013 | 0.98 [0.72, 1.33] | 0.882 |
| **Division** |  |  |  |  |  |  |  |  |  |
| Barisal | Ref. |  |  |  |  | Ref. |  | Ref. |  |
| Chattogram | 0.66 [0.42, 1.04] | 0.074 |  |  |  | 0.64 [0.41, 1.01] | 0.053 | 0.46 [0.28, 0.74] | 0.001 |
| Dhaka | 0.93 [0.59, 1.47] | 0.761 |  |  |  | 0.88 [0.56, 1.37] | 0.563 | 0.64 [0.40, 1.04] | 0.072 |
| Khulna | 0.99 [0.61, 1.62] | 0.982 |  |  |  | 0.97 [0.59, 1.58] | 0.906 | 0.71 [0.42, 1.21] | 0.212 |
| Rajshahi | 0.96 [0.58, 1.6] | 0.871 |  |  |  | 0.95 [0.57, 1.58] | 0.846 | 0.79 [0.45, 1.38] | 0.408 |
| Rangpur | 1.32 [0.81, 2.15] | 0.268 |  |  |  | 1.32 [0.81, 2.15] | 0.258 | 1.22 [0.72, 2.06] | 0.462 |
| Sylhet | 0.53 [0.32, 0.87] | 0.012 |  |  |  | 0.53 [0.32, 0.88] | 0.014 | 0.47 [0.27, 0.84] | 0.011 |
| **Random-effects (measures of variations)** |  |  |  |  |  |  |  |  |  |
| Cluster level variance (SE) |  |  | 0.51 (0.12) | 0.52 (0.13) |  | 0.44 (0.11) |  | 0.45 (0.12) |  |
| P‐value |  |  |  | <0.001 |  | <0.001 |  | <0.001 |  |
| PCV (%) |  |  | Ref. | -1.96 |  | 13.70 |  | 11.80 |  |
| ICC (%) |  |  | 13.30 | 13.70 |  | 11.70 |  | 12.00 |  |
| MOR |  |  | 1.97 | 1.99 |  | 1.88 |  | 1.90 |  |
| **Fit criteria (model diagnostics)** |  |  |  |  |  |  |  |  |  |
| Loglikelihood |  |  | -1389.24 | -1232.23 |  | -1377.60 |  | -1221.21 |  |
| AIC |  |  | 2782.48 | 2508.46 |  | 2773.20 |  | 2500.41 |  |
| BIC |  |  | 2793.94 | 2634.60 |  | 2824.80 |  | 2666.69 |  |

COR, crude odds ratio; CI, confidence interval; AOR, adjusted odds ratio; Ref, reference; SE, standard error, PCV, proportional change in variance; ICC, intra-cluster correlation coefficient; MOR, median odds ratio; AIC, Akaike’s information criterion; BIC, Bayesian information criterion.

**Table S4.** Factors associated with the consumption of ASF among children 6 to 23 months of age in 2017/18.

| **Characteristics** | **Unadjusted model** | | **Model I** | **Model II** | | **Model III** | | **Model IV** | |
| --- | --- | --- | --- | --- | --- | --- | --- | --- | --- |
|  | **COR [95%CI]** | **P value** |  | **AOR [95%CI]** | **P value** | **AOR [95%CI]** | **P value** | **AOR [95%CI]** | **P value** |
| **Child age in months** |  |  |  |  |  |  |  |  |  |
| 6–8 | Ref. |  |  | Ref. |  |  |  | Ref. |  |
| 9–11 | 2.10 [1.47, 3.00] | <0.001 |  | 2.13 [1.48, 3.08] | <0.001 |  |  | 2.14 [1.49, 3.07] | <0.001 |
| 12–17 | 3.94 [2.82, 5.49] | <0.001 |  | 3.79 [2.73, 5.26] | <0.001 |  |  | 3.87 [2.79, 5.36] | <0.001 |
| 18–23 | 6.81 [4.67, 9.91] | <0.001 |  | 6.57 [4.50, 9.60] | <0.001 |  |  | 6.62 [4.52, 9.70] | <0.001 |
| **Birth order** |  |  |  |  |  |  |  |  |  |
| First |  |  |  | Ref. |  |  |  | Ref. |  |
| 2 to 3 | 0.77 [0.59, 1.01] | 0.059 |  | 0.81 [0.59, 1.10] | 0.169 |  |  | 0.83 [0.61, 1.13] | 0.240 |
| ≥4 | 0.48 [0.33, 0.69] | <0.001 |  | 0.72 [0.46, 1.13] | 0.154 |  |  | 0.77 [0.49, 1.22] | 0.267 |
| **Recent morbidity** |  |  |  |  |  |  |  |  |  |
| No | Ref. |  |  | Ref. |  |  |  | Ref. |  |
| Yes | 0.82 [0.65, 1.03] | 0.089 |  | 0.88 [0.69, 1.13] | 0.314 |  |  | 0.87 [0.68, 1.11] | 0.264 |
| **Maternal education** |  |  |  |  |  |  |  |  |  |
| No formal education | Ref. |  |  | Ref. |  |  |  | Ref. |  |
| Formal education | 3.48 [2.25, 5.37] | <0.001 |  | 2.79 [1.69, 4.61] | <0.001 |  |  | 2.74 [1.65, 4.55] | <0.001 |
| **Paternal education** |  |  |  |  |  |  |  |  |  |
| No formal education | Ref. |  |  | Ref. |  |  |  | Ref. |  |
| Formal education | 1.66 [1.25, 2.20] | <0.001 |  | 1.11 [0.78, 1.57] | 0.554 |  |  | 1.09 [0.77, 1.54] | 0.624 |
| **Maternal occupation** |  |  |  |  |  |  |  |  |  |
| Unemployed | Ref. |  |  | Ref. |  |  |  | Ref. |  |
| Agricultural | 0.97 [0.73, 1.28] | 0.830 |  | 1.18 [0.87, 1.60] | 0.276 |  |  | 1.07 [0.79, 1.45] | 0.682 |
| Non-agricultural | 1.43 [0.96, 2.12] | 0.081 |  | 1.48 [0.97, 2.25] | 0.069 |  |  | 1.42 [0.93, 2.15] | 0.100 |
| **Maternal pregnancy status** |  |  |  |  |  |  |  |  |  |
| No | Ref. |  |  | Ref. |  |  |  | Ref. |  |
| Yes | 6.51 [2.36, 17.97] | <0.001 |  | 4.36 [1.53, 12.42] | 0.006 |  |  | 4.87 [1.76, 13.47] | 0.002 |
| **Frequency of ANC** |  |  |  |  |  |  |  |  |  |
| No | Ref. |  |  | Ref. |  |  |  | Ref. |  |
| 1-3 times | 1.37 [0.93, 2.03] | 0.111 |  | 1.09 [0.71, 1.68] | 0.677 |  |  | 1.04 [0.68, 1.60] | 0.857 |
| ≥4 times | 2.31 [1.53, 3.46] | <0.001 |  | 1.51 [0.95, 2.38] | 0.079 |  |  | 1.36 [0.85, 2.17] | 0.204 |
| **Maternal age of first birth** |  |  |  |  |  |  |  |  |  |
| <20 years | Ref. |  |  | Ref. |  |  |  | Ref. |  |
| ≥20 years | 1.24 [0.96, 1.60] | 0.104 |  | 0.97 [0.73, 1.29] | 0.828 |  |  | 0.99 [0.74, 1.31] | 0.930 |
| **Maternal access to media** |  |  |  |  |  |  |  |  |  |
| No | Ref. |  |  | Ref. |  |  |  | Ref. |  |
| Yes | 1.58 [1.26, 1.98] | <0.001 |  | 1.14 [0.85, 1.54] | 0.377 |  |  | 1.08 [0.80, 1.46] | 0.629 |
| **Family size** |  |  |  |  |  |  |  |  |  |
| ≤4 members | Ref. |  |  | Ref. |  |  |  | Ref. |  |
| ≥5 members | 0.69 [0.53, 0.90] | 0.007 |  | 0.72 [0.53, 0.96] | 0.024 |  |  | 0.74 [0.55, 0.99] | 0.043 |
| **Wealth index** |  |  |  |  |  |  |  |  |  |
| Poorest | Ref. |  |  | Ref. |  |  |  | Ref. |  |
| Poorer | 1.30 [0.93, 1.82] | 0.121 |  | 1.19 [0.82, 1.72] | 0.356 |  |  | 1.21 [0.83, 1.75] | 0.327 |
| Middle | 1.72 [1.20, 2.46] | 0.003 |  | 1.4 [0.93, 2.12] | 0.108 |  |  | 1.46 [0.97, 2.21] | 0.070 |
| Richer | 1.54 [1.08, 2.19] | 0.016 |  | 1.23 [0.81, 1.86] | 0.325 |  |  | 1.38 [0.91, 2.11] | 0.130 |
| Richest | 2.68 [1.75, 4.11] | <0.001 |  | 1.86 [1.14, 3.05] | 0.014 |  |  | 2.21 [1.31, 3.72] | 0.003 |
| **Place of residence** |  |  |  |  |  |  |  |  |  |
| Urban | Ref. |  |  |  |  | Ref. |  | Ref. |  |
| Rural | 0.83 [0.63, 1.08] | 0.168 |  |  |  | 0.84 [0.64, 1.09] | 0.182 | 1.20 [0.88, 1.63] | 0.255 |
| **Division** |  |  |  |  |  |  |  |  |  |
| Barisal | Ref. |  |  |  |  | Ref. |  | Ref. |  |
| Chattogram | 0.98 [0.63, 1.54] | 0.938 |  |  |  | 0.97 [0.62, 1.52] | 0.885 | 0.86 [0.55, 1.35] | 0.509 |
| Dhaka | 1.35 [0.88, 2.07] | 0.173 |  |  |  | 1.3 [0.85, 1.98] | 0.234 | 1.16 [0.75, 1.78] | 0.508 |
| Khulna | 1.48 [0.91, 2.42] | 0.114 |  |  |  | 1.47 [0.90, 2.41] | 0.122 | 1.28 [0.78, 2.09] | 0.335 |
| Rajshahi | 1.4 [0.83, 2.36] | 0.212 |  |  |  | 1.39 [0.82, 2.36] | 0.215 | 1.18 [0.69, 2.02] | 0.557 |
| Rangpur | 1.79 [1.06, 3.03] | 0.029 |  |  |  | 1.81 [1.07, 3.06] | 0.027 | 1.66 [0.96, 2.88] | 0.070 |
| Sylhet | 0.71 [0.44, 1.14] | 0.156 |  |  |  | 0.71 [0.44, 1.15] | 0.160 | 0.65 [0.39, 1.09] | 0.105 |
| **Random-effects (measures of variations)** |  |  |  |  |  |  |  |  |  |
| Cluster level variance (SE) |  |  | 0.40 (0.13) | 0.38 (0.16) |  | 0.33 (0.12) |  | 0.34 (0.15) |  |
| P‐value |  |  |  | <0.001 |  | <0.001 |  | <0.001 |  |
| PCV (%) |  |  | Ref. | 5.00 |  | 17.50 |  | 15.00 |  |
| ICC (%) |  |  | 10.90 | 10.30 |  | 9.20 |  | 9.30 |  |
| MOR |  |  | 1.82 | 1.80 |  | 1.73 |  | 1.75 |  |
| **Fit criteria (model diagnostics)** |  |  |  |  |  |  |  |  |  |
| Loglikelihood |  |  | -1219.87 | -1089.79 |  | -1209.76 |  | -1082.45 |  |
| AIC |  |  | 2443.73 | 2223.58 |  | 2437.51 |  | 2222.90 |  |
| BIC |  |  | 2455.28 | 2350.57 |  | 2489.46 |  | 2390.29 |  |

COR, crude odds ratio; CI, confidence interval; AOR, adjusted odds ratio; Ref, reference; SE, standard error, PCV, proportional change in variance; ICC, intra-cluster correlation coefficient; MOR, median odds ratio; AIC, Akaike’s information criterion; BIC, Bayesian information criterion.

**Table S5.** Factors associated with the consumption of ASF among children 6 to 23 months of age in 2022.

| **Characteristics** | **Unadjusted model** | | **Model I** | **Model II** | | **Model III** | | **Model IV** | |
| --- | --- | --- | --- | --- | --- | --- | --- | --- | --- |
|  | **COR [95%CI]** | **P value** |  | **AOR [95%CI]** | **P value** | **AOR [95%CI]** | **P value** | **AOR [95%CI]** | **P value** |
| **Child age in months** |  |  |  |  |  |  |  |  |  |
| 6–8 | Ref. |  |  | Ref. |  |  |  | Ref. |  |
| 9–11 | 1.99 [1.38, 2.86] | <0.001 |  | 1.94 [1.35, 2.80] | <0.001 |  |  | 1.97 [1.36, 2.85] | <0.001 |
| 12–17 | 3.61 [2.58, 5.03] | <0.001 |  | 3.34 [2.37, 4.70] | <0.001 |  |  | 3.36 [2.39, 4.72] | <0.001 |
| 18–23 | 5.23 [3.72, 7.34] | <0.001 |  | 5.53 [3.90, 7.83] | <0.001 |  |  | 5.58 [3.95, 7.88] | <0.001 |
| **Birth order** |  |  |  |  |  |  |  |  |  |
| First | Ref. |  |  | Ref. |  |  |  | Ref. |  |
| 2 to 3 | 0.93 [0.74, 1.19] | 0.578 |  | 0.89 [0.66, 1.20] | 0.441 |  |  | 0.92 [0.68, 1.25] | 0.604 |
| ≥4 | 0.68 [0.46, 1.01] | 0.058 |  | 0.84 [0.49, 1.43] | 0.517 |  |  | 0.96 [0.56, 1.66] | 0.890 |
| **Recent morbidity** |  |  |  |  |  |  |  |  |  |
| No | Ref. |  |  | Ref. |  |  |  | Ref. |  |
| Yes | 0.78 [0.62, 0.97] | 0.026 |  | 0.78 [0.62, 0.98] | 0.035 |  |  | 0.80 [0.64, 1.01] | 0.057 |
| **Maternal age in years** |  |  |  |  |  |  |  |  |  |
| 14-19 | Ref. |  |  | Ref. |  |  |  | Ref. |  |
| 20-29 | 1.29 [0.93, 1.79] | 0.129 |  | 1.23 [0.83, 1.82] | 0.309 |  |  | 1.28 [0.86, 1.91] | 0.220 |
| 30-39 | 1.44 [0.98, 2.10] | 0.062 |  | 1.37 [0.81, 2.32] | 0.230 |  |  | 1.33 [0.78, 2.24] | 0.293 |
| 40-49 | 0.55 [0.22, 1.37] | 0.202 |  | 0.82 [0.32, 2.10] | 0.680 |  |  | 0.83 [0.33, 2.11] | 0.700 |
| **Maternal education** |  |  |  |  |  |  |  |  |  |
| No formal education | Ref. |  |  | Ref. |  |  |  | Ref. |  |
| Formal education | 2.05 [1.23, 3.43] | 0.006 |  | 1.36 [0.78, 2.38] | 0.281 |  |  | 1.43 [0.83, 2.46] | 0.200 |
| **Paternal education** |  |  |  |  |  |  |  |  |  |
| No formal education | Ref. |  |  | Ref. |  |  |  | Ref. |  |
| Formal education | 2.19 [1.65, 2.90] | <0.001 |  | 1.58 [1.16, 2.15] | 0.004 |  |  | 1.56 [1.14, 2.12] | 0.005 |
| **Maternal occupation** |  |  |  |  |  |  |  |  |  |
| Unemployed | Ref. |  |  | Ref. |  |  |  | Ref. |  |
| Agricultural | 0.99 [0.73, 1.34] | 0.948 |  | 1.19 [0.86, 1.65] | 0.294 |  |  | 1.12 [0.81, 1.55] | 0.486 |
| Non-agricultural | 1.48 [0.96, 2.27] | 0.076 |  | 1.37 [0.86, 2.18] | 0.189 |  |  | 1.27 [0.80, 2.03] | 0.315 |
| **Paternal occupation** |  |  |  |  |  |  |  |  |  |
| Unemployed | Ref. |  |  | Ref. |  |  |  | Ref. |  |
| Agricultural | 1.25 [0.54, 2.90] | 0.609 |  | 1.84 [0.76, 4.48] | 0.178 |  |  | 1.32 [0.54, 3.24] | 0.546 |
| Non-agricultural | 1.72 [0.76, 3.90] | 0.191 |  | 2.13 [0.91, 5.01] | 0.083 |  |  | 1.69 [0.70, 4.03] | 0.241 |
| **Maternal pregnancy status** |  |  |  |  |  |  |  |  |  |
| No | Ref. |  |  | Ref. |  |  |  | Ref. |  |
| Yes | 2.06 [1.05, 4.05] | 0.036 |  | 1.84 [0.91, 3.70] | 0.088 |  |  | 2.04 [0.98, 4.23] | 0.057 |
| **Frequency of ANC** |  |  |  |  |  |  |  |  |  |
| No | Ref. |  |  | Ref. |  |  |  | Ref. |  |
| 1-3 times | 1.88 [1.26, 2.79] | 0.002 |  | 1.67 [1.10, 2.52] | 0.016 |  |  | 1.59 [1.04, 2.43] | 0.031 |
| ≥4 times | 2.57 [1.68, 3.91] | <0.001 |  | 2.13 [1.35, 3.34] | 0.001 |  |  | 2.03 [1.29, 3.20] | 0.002 |
| **Maternal age of first birth** |  |  |  |  |  |  |  |  |  |
| <20 years | Ref. |  |  | Ref. |  |  |  | Ref. |  |
| ≥20 years | 0.14 [0.94, 1.49] | 0.152 |  | 0.93 [0.70, 1.24] | 0.634 |  |  | 1.01 [0.76, 1.35] | 0.935 |
| **Maternal access to media** |  |  |  |  |  |  |  |  |  |
| No | Ref. |  |  | Ref. |  |  |  | Ref. |  |
| Yes | 1.64 [1.28, 2.10] | <0.001 |  | 1.37 [1.05, 1.80] | 0.021 |  |  | 1.24 [0.94, 1.62] | 0.128 |
| **Family size** |  |  |  |  |  |  |  |  |  |
| ≤4 members | Ref. |  |  | Ref. |  |  |  | Ref. |  |
| ≥5 members | 1.18 [0.95, 1.48] | 0.136 |  | 1.29 [1.01, 1.64] | 0.041 |  |  | 1.41 [1.11, 1.80] | 0.006 |
| **Wealth index** |  |  |  |  |  |  |  |  |  |
| Poorest | Ref. |  |  | Ref. |  |  |  | Ref. |  |
| Poorer | 1.20 [0.89, 1.64] | 0.236 |  | 1.00 [0.72, 1.38] | 0.979 |  |  | 0.97 [0.70, 1.34] | 0.852 |
| Middle | 1.83 [1.29, 2.60] | 0.001 |  | 1.61 [1.11, 2.33] | 0.011 |  |  | 1.53 [1.05, 2.23] | 0.026 |
| Richer | 1.79 [1.20, 2.68] | 0.004 |  | 1.37 [0.91, 2.07] | 0.131 |  |  | 1.32 [0.87, 2.01] | 0.189 |
| Richest | 3.23 [2.19, 4.76] | <0.001 |  | 2.19 [1.38, 3.48] | 0.001 |  |  | 2.13 [1.30, 3.48] | 0.003 |
| **Place of residence** |  |  |  |  |  |  |  |  |  |
| Urban | Ref. |  |  |  |  | Ref. |  | Ref. |  |
| Rural | 0.75 [0.57, 0.98] | 0.036 |  |  |  | 0.81 [0.63, 1.05] | 0.107 | 1.13 [0.84, 1.51] | 0.420 |
| **Division** |  |  |  |  |  |  |  |  |  |
| Barisal | Ref. |  |  |  |  | Ref. |  | Ref. |  |
| Chattogram | 0.74 [0.47, 1.17] | 0.203 |  |  |  | 0.72 [0.46, 1.14] | 0.165 | 0.60 [0.37, 0.97] | 0.038 |
| Dhaka | 1.69 [1.10, 2.59] | 0.017 |  |  |  | 1.61 [1.05, 2.46] | 0.029 | 1.38 [0.88, 2.17] | 0.164 |
| Khulna | 2.25 [1.33, 3.80] | 0.002 |  |  |  | 2.22 [1.31, 3.76] | 0.003 | 1.79 [1.04, 3.10] | 0.037 |
| Rajshahi | 2.09 [1.21, 3.62] | 0.008 |  |  |  | 2.08 [1.20, 3.59] | 0.009 | 1.84 [1.05, 3.22] | 0.032 |
| Rangpur | 1.01 [0.63, 1.61] | 0.965 |  |  |  | 1.01 [0.63, 1.62] | 0.956 | 0.92 [0.56, 1.51] | 0.734 |
| Sylhet | 0.46 [0.30, 0.72] | 0.001 |  |  |  | 0.46 [0.30, 0.72] | 0.001 | 0.39 [0.24, 0.64] | <0.001 |
| **Random-effects (measures of variations)** |  |  |  |  |  |  |  |  |  |
| Cluster level variance (SE) |  |  | 0.69 (0.14) | 0.57 (0.14) |  | 0.47 (0.14) |  | 0.41 (0.13) |  |
| P‐value |  |  |  | <0.001 |  | <0.001 |  | <0.001 |  |
| PCV (%) |  |  | Ref. | 17.40 |  | 31.90 |  | 40.60 |  |
| ICC (%) |  |  | 17.40 | 14.80 |  | 12.40 |  | 11.10 |  |
| MOR |  |  | 2.20 | 2.05 |  | 1.92 |  | 1.84 |  |
| **Fit criteria (model diagnostics)** |  |  |  |  |  |  |  |  |  |
| Loglikelihood |  |  | -1394.36 | -1261.56 |  | -1359.42 |  | -1231.89 |  |
| AIC |  |  | 2792.72 | 2577.12 |  | 2736.85 |  | 2531.78 |  |
| BIC |  |  | 2804.31 | 2733.63 |  | 2789.02 |  | 2728.88 |  |

COR, crude odds ratio; CI, confidence interval; AOR, adjusted odds ratio; Ref, reference; SE, standard error, PCV, proportional change in variance; ICC, intra-cluster correlation coefficient; MOR, median odds ratio; AIC, Akaike’s information criterion; BIC, Bayesian information criterion.
